# Supplementary material for: Repeated Viewing of a Narrative Movie Changes Event Timescales in The Brain
Source: bioRxiv. 2025 Sep 1:2025.08.27.672403. Preprint. [Version 1] doi: 10.1101/2025.08.27.672403 (PMC12424988; doi:10.1101/2025.08.27.672403)
Supplement: Supplement 1 [file NIHPP2025.08.27.672403v1-supplement-1.pdf]

bioRxiv preprint doi: https://doi.org/10.1101/2025.08.27.672403; this version posted September 1, 2025. The copyright holder for this preprint (which was not certified by peer review) is the author/funder, who has granted bioRxiv a license to display the preprint in perpetuity. It is made available under aCC-BY 4.0 International license.

Supplementary Figures

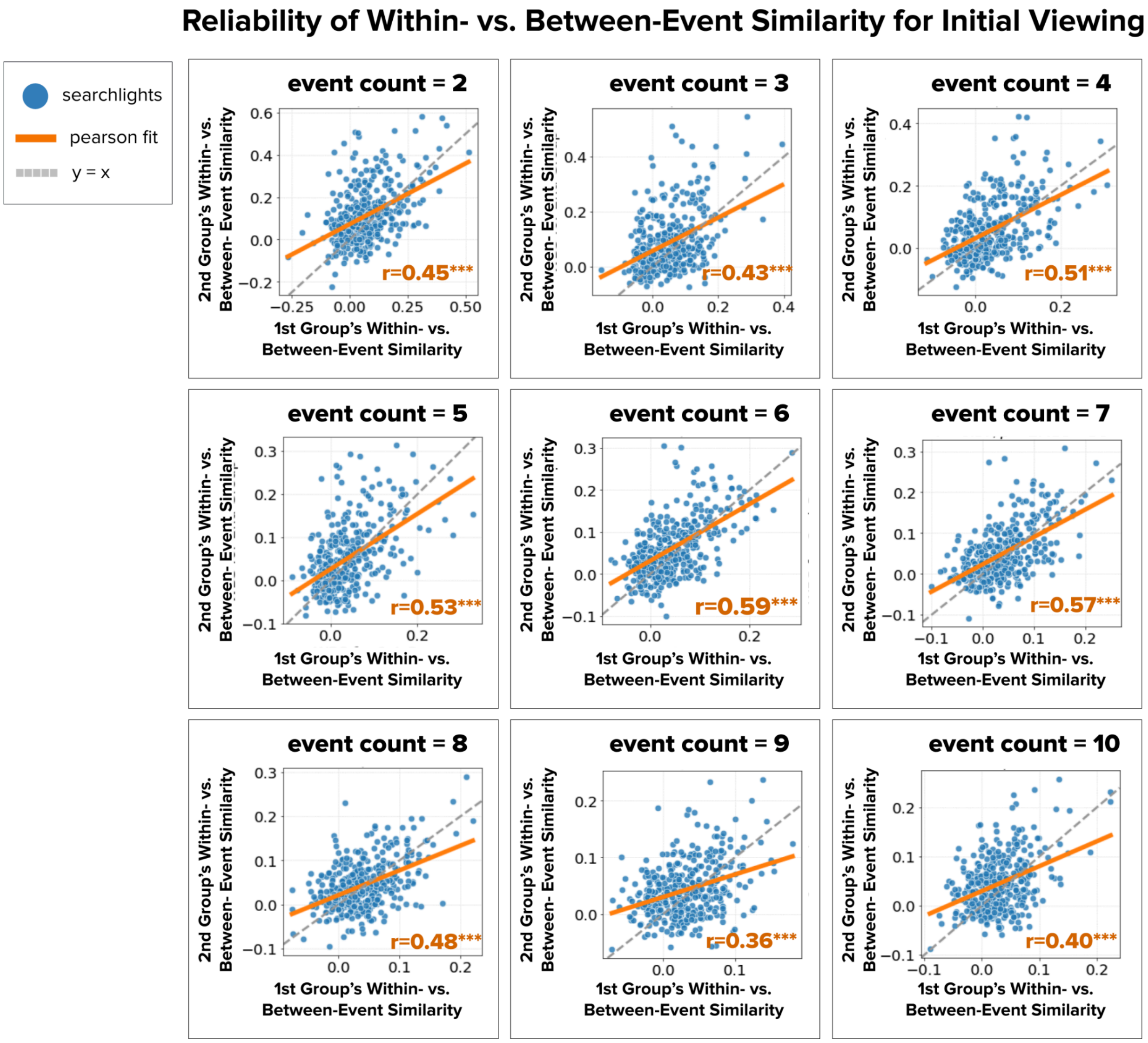

Supplementary Figure 1. Validation of Within- vs. Between-Event Similarity (Intact Clip). To assess the reliability of the Within- vs. Between-Event Similarity measure during participants' initial viewing of the movie clip, we examined how correlated this measure was across searchlights for two independent participant samples. Participants were first randomly divided into two independent groups of 15 participants each. Then, within each group separately, participants were randomly divided into training and testing subsets. We identified HMM event boundaries for each searchlight in the training subset and then used those boundaries to compute Within- vs. Between-Event Similarity in the testing subset. Training and testing subsets were then swapped, and results were averaged across five different participant splits within each independent group. Finally, we examined the correlation in Within- vs. Between-Event Similarity values across the two independent groups. Reliability for Within- vs. Between-Event Similarity values was consistently high across all event counts, demonstrating that this measure is relatively stable across subsets of participants.

bioRxiv preprint doi: https://doi.org/10.1101/2025.08.27.672403; this version posted September 1, 2025. The copyright holder for this preprint (which was not certified by peer review) is the author/funder, who has granted bioRxiv a license to display the preprint in perpetuity. It is made available under aCC-BY 4.0 International license.

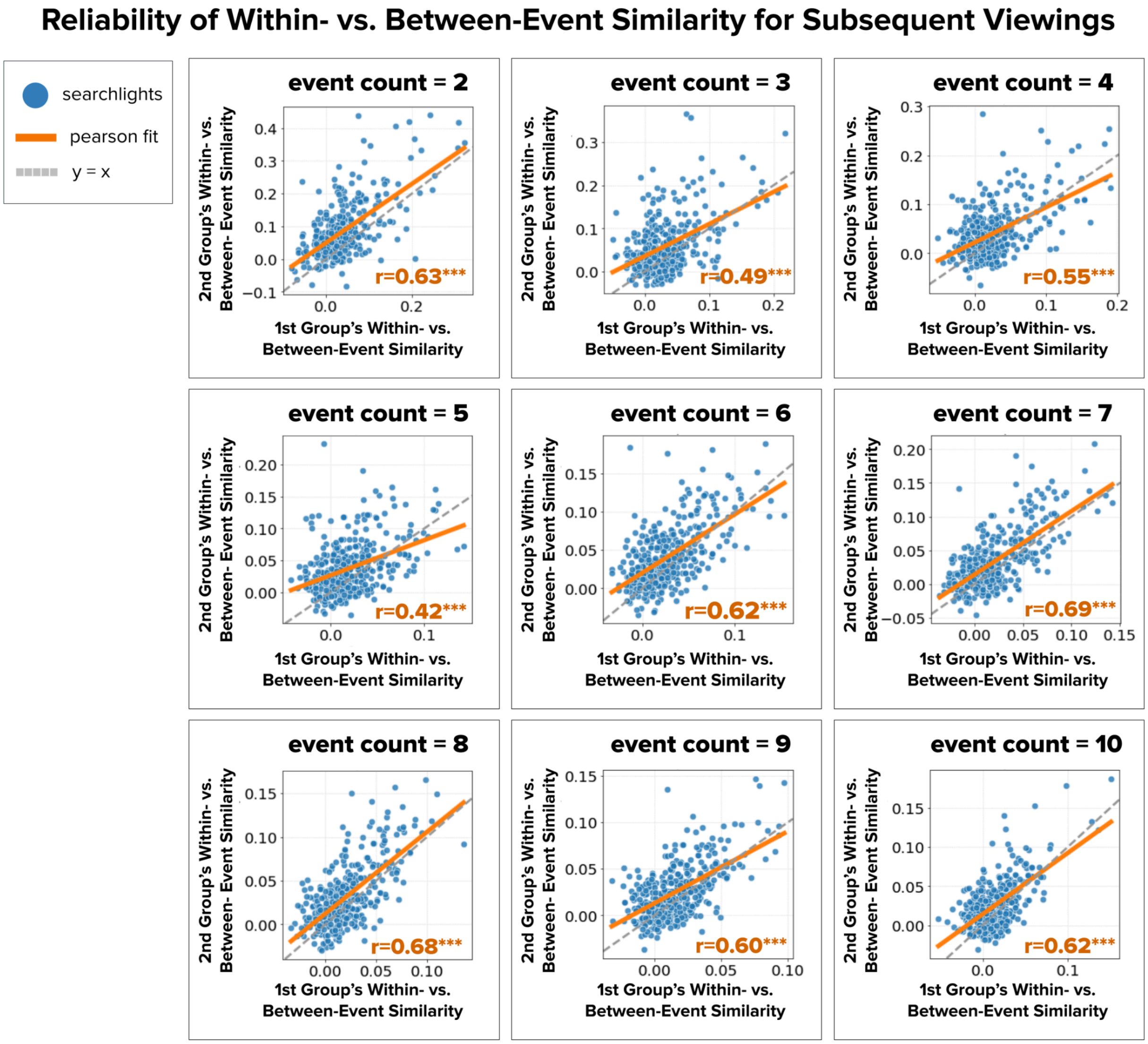

Supplementary Figure 2. Validation for Subsequent Viewings (Intact Clip). The same split-half reliability procedure was repeated for participants' subsequent viewings of the narrative. Results again showed strong reliability across all event counts, indicating that the Within- vs. Between-Event Similarity measure remains robust across repeated viewings.
